# Pulmonary Vein Isolation by High Intensity Focused Ultrasound

**Published:** 2007-04-01

**Authors:** Boris Schmidt, KR Julian Chun, Karl-Heinz Kuck, Matthias Antz

**Affiliations:** Asklepios Klinik St. Georg, Department of Cardiology, Lohmuhlenstr. 5, 22099 Hamburg, Germany

**Keywords:** Atrial fibrillation, pulmonary vein isolation, catheter ablation, high intensity focused ultrasound, phrenic nerve palsy

## Abstract

Pulmonary vein isolation (PVI) using radiofrequency current (RFC) ablation is a potentially curative treatment option for patients with atrial fibrillation (AF). The shortcomings of the RFC technology (technically challenging, long procedure times, complications) steadily kindle the interest in new energy sources and catheter designs. High intensity focused ultrasound (HIFU) has the ability to precisely focus ultrasound waves in a defined area with a high energy density. HIFU balloon catheters (BC) positioned at the PV ostia appear to be an ideal tool to transmit the ablation energy in a circumferential manner to the PV ostia and may therefore bear substantial advantage over conventional ablation catheters in PVI procedures.

In clinical trials the HIFU BC has shown promising success rates similar to RFC catheter ablation for PVI in patients with AF. However, procedure times are still long and serious complications have been observed. Therefore, it may be a valuable alternative to the conventional techniques in selected patients but further clinical trials have to be initiated.

## Introduction

The discovery of electrical triggers in the pulmonary veins (PV) inducing and entertaining atrial fibrillation (AF) initiated the development of new, potentially curative treatment strategies for patients with drug refractory paroxysmal AF. Electrical PV isolation (PVI) by disconnection of muscular sleeves extending from the left atrium (LA) into the PV is currently the most promising approach used in ablation procedures. Radiofrequency current (RFC) is the most commonly used energy source to create ablation lesions. In order to achieve complete PVI the muscular sleeves are either targeted focally (known as segmental ablation) or ablated by circumferential circular lesions at some distance to the veno-atrial junction [1-3].

Extensive research data and experience is available regarding RFC and is therefore exclusively used for PVI in most electrophysiological (EP) laboratories. However, with RFC it can be technically challenging to create continuous circumferential linear lesions even if the "drag and burn" ablation strategy is applied. This results in time consuming ablation procedures increasing the discomfort and risk for complications for both, the patient and the physician.

In addition, it bears the potential risk for rare but sometimes fatal complications such as PV stenosis (1-5%), stroke and atrio-esophageal fistula formation [4,5]. Additional disadvantages of RFC include the risk of thrombus formation on the ablation electrode or perforations due to steam pops caused by tissue overheating.

The shortcomings of the RFC technology steadily kindle the interest in new energy sources and catheter designs. Ideally, the new energy should be safe and effective, e.g. create transmyocardial circumferential lesions with a single application.

High intensity focused ultrasound (HIFU) was originally developed as an extracorporeal tool for the treatment of tumors. The ability to precisely focus ultrasound waves in a defined area with a high energy density appeared to be an ideal technology to destroy tissue with only minimal collateral damage.

Balloon mounted catheters positioned at the PV ostia appear to have an ideal shape to transmit the ablation energy in a circumferential manner to the neighbored tissue and may therefore bear substantial advantage over conventional ablation catheters in PVI procedures.

In the following review article the concept, technical considerations and first animal and clinical data using balloon mounted HIFU catheters for PVI will be presented.

## Technical concept of HIFU

HIFU relies on the basic concepts of conventional ultrasound. Mechanical vibrations above the threshold of the human hearing are called ultrasound. Ultrasound waves may propagate through living tissue and fluids without causing any harm to the cells [4]. By focusing highly energetic ultrasound waves to a well defined volume, local heat rise (usually >56°C and up to 80°C) occurs and causes rapid tissue necrosis by coagulative necrosis. Fortunately, a steep temperature gradient is observed between the focus and the surrounding tissue allowing for the production of sharply demarcated lesions and reducing collateral damage [7].

Another mechanism by which HIFU destroys tissue is called acoustic cavitation [8]. This process is based on vibration of cellular structures causing local hyperthermia and mechanical stress by bubble formation due to rapid changes in local pressure leading to cell death.

In general, extracorporeal and invasive catheter mounted devices are commercially available. Since extracorporeal tools are currently not clinically used for cardiac ablation the authors will focus on catheter mounted HIFU devices.

Ultrasound transducers mounted on angiographic catheters were initially used to investigate the feasibility of creating focal lesions in ventricular myocardium [9]. In order to perform more complex ablations such as PVI procedures a further refinement of the catheter technology was mandatory. Therefore, balloon mounted ultrasound ablation catheters were developed. The basic principle of these balloon catheters (BC) is an ultrasound crystal housed in a fluid filled balloon. The Atrionix BC (Atrionix, Inc.) is a non-steerable over-the-wire device and consists of a single balloon emitting non focused ultrasound transradially [10].

**Figure 1:**
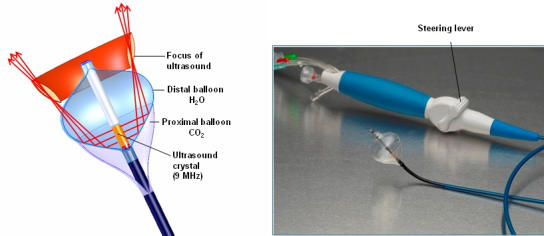
Basic principle of the HIFU BC (left panel) and a steerable HIFU-BC (right panel). See text for details.

Oppositely, the HIFU ablation catheter (ProRhythm, Inc.) consists of a non-compliant distal balloon, which is filled with a mixture of water and contrast media (6:1 ratio) and an integrated 9 MHz ultrasound crystal. Proximal, a second non-compliant balloon, filled with carbon dioxide, forms a parabolic surface at the base of the distal balloon. Thereby, the ultrasound waves are reflected in the forward direction, focusing a ring of ultrasound energy (sonicating ring) ~4 mm distally to the balloon surface (Figure 1). The new generation of HIFU BC are steerable through a pull wire mechanism integrated in the handle of the catheter. Three different balloon sizes are available: a 24 mm diameter balloon (20 mm sonicating ring diameter), 27 mm diameter balloon (sonicating ring 25 mm diameter) and a 32 mm diameter balloon (30 mm sonication ring diameter). The catheter has a central lumen used for PV angiography (distal to the balloon) and for insertion of a guide wire (0.035 inch) supporting the navigation of the catheter.

## Lesion formation

A major advantage of HIFU is that no direct tissue contact is needed for the generation of the ablation lesion. In several pre-clinical studies it was demonstrated that lesions were formed as ultrasound waves are focused in a precisely defined zone leading to a high focal energy density [11]. Consequently, tissue damage is caused by heat reaching maximal tissue temperatures of 80° C rather than tissue disruption. Lesion volume depends on sonication time and on the initial tissue temperature as well as on the amount of acoustic power [12,13]. He and colleagues demonstrated that transmural lesions (up to 9mm depth) can be achieved in ventricular myocardium using catheter mounted ultrasound transducers in beating canine left ventricles [9,14]. Ablation lesions were usually well demarcated without any collateral damage to the surrounding tissue [15].

The balloon mounted HIFU catheter was designed to create circular ablation lesions as desired in PVI procedures in order to overcome the above mentioned limitations observed for RFC. According to the balloon shape, a circular ablation lesion can be formed by a single application of HIFU. In 40-90 sec a complete transmural lesion can be achieved depending on the size of the balloon.

## Animal studies

The first experience with HIFU in a beating heart was reported by Zimmer et al [14,16]. HIFU guided ablations were performed in beating canine hearts using catheter mounted HIFU transducers. Later, Strickberger and colleagues performed complete atrio-ventricular (AV) junction ablation in ten anesthetized canines [17]. After thoracotomy a 7.0-MHz diagnostic ultrasound probe (Diasonics VST Master Series, Diasonics/Vingmed Ultrasound Inc.) was affixed to a spherically focused 1.4 MHz HIFU transducer. From outside the heart the maximum ultrasound intensity for ablation (2.8 kW/cm^2^) was delivered to the AV junction for a total of 30 sec. Complete AV block was achieved in each of the 10 dogs with a mean of 6.5 (range, 3 to 21) HIFU applications. The lesions were distinct and showed a depth of 6.7 ± 3.6 mm, a length of 5.7 ± 2.5 mm, and a width of 4.7 ± 1.8 mm.

Nakagawa and colleagues investigated the effects of an intracardiac HIFU BC. In a pre-clinical canine study, acute PVI was achieved using the HIFU BC. PVI persisted in 88% at a follow up study performed after 1 week to 3 months [11]. No thrombus formation at the ablation site was observed and lesions were formed even at sites with a lack of balloon to tissue contact. In this trial no PV stenosis occurred.

## Clinical studies

The first report on PVI by ultrasound ablation in humans was published by Natale and co-workers [10]. Fifteen patients with AF refractory to medication underwent PVI with a non-steerable BC using non-focused ultrasound (Atrionix, Inc.). The catheter was navigated over-the-wire, enabling access of the right inferior PV (RIPV) in only one patient. In the remaining patients ultrasound ablation was performed only at the right superior and both left PVs. The median number of lesions per patient required to isolate 1 pulmonary vein was 4 (range, 1 to 29). The mean procedure time was 224 ± 89 minutes (range, 135 to 360 minutes). The mean fluoroscopic time was 62 ± 39 minutes (range 37 to 120 minutes). After ablation, no evidence of narrowing was seen with repeat venography or follow-up computed tomography scan. However, 2 patients experienced thrombembolic complications (1 Stroke, 1 transient myocardial ischemia by air embolism) and 1 patient experienced right sided phrenic nerve (PN) palsy. After a mean follow-up of 35 ± 6 weeks, one third of patients had AF recurrences.

In collaboration with the working group from Oklahoma we reported on the results of PVI with the non-steerable HIFU BC (ProRhythm, Inc.) in 27 patients with paroxysmal (19 patients) or persistent (8 patients) AF [18].  PV antrum isolation was attempted using HIFU BC in 78/104 PVs (25/27 RSPVs, all 23LSPVs, all 23LIPVs, all 4 left common trunks and 3/27RIPVs). HIFU BC successfully isolated 68 (87%) of the 78 PV ostia with 1-26 (median 3) HIFU applications. The complications included transient bleeding from a distal branch of the left superior PV resulting from guide wire manipulation in one patient and right PN injury in another patient. No PV stenosis (>50% narrowing) and no LA-esophageal fistula was observed. At the 12 month follow-up, 16/27 (59%) patients were free of symptomatic AF episodes.

In a consecutive study 15 pts (7 female) with paroxysmal AF were treated with the steerable HIFU BC (ProRhythm Inc.) [19]. The improved steerability increased the success rate for complete PVI to 89% (41/46 PVs; 2). After a median follow-up of 387 days (range 120 - 424 days) 7/12 (58%) of patients were free of AF without antiarrhythmic drugs and in 2 patients only a single AF episode was documented, resulting in an overall chronic success of 75%. Unfortunately, 2 pts experienced right sided PN palsy, which had not resolved after 12 months.

Using the steerable balloon all PV (even the RIPV) could be accessed. Meticulous mapping of the ablation lesions demonstrated a rather ostial location. As revealed by 3D reconstructions of magnetic resonance angiographies of LA and PV the left sided PV often display a flat anatomy, resulting from a pronounced mismatch of the maximal and minimal diameters [20]. In addition, the LPV are separated from the LAA only by a narrow myocardial ridge. It became evident that by using HIFU BC it remains challenging to achieve complete PVI for the LPV because the balloon overlaps into the LAA hindering sonication of the ridge. Undersized balloons were frequently used to overcome this limitation.

## Limitations

In general, HIFU BC ablation can be considered in all patients scheduled for PVI. However, patients with common PV ostia with diameters > 30mm may not be eligible because the maximal available balloon size is only 30mm.

Today, no HIFU catheter is available which can be used for linear (instead of circular) ablation. Thus, if additional linear lesions are required, as proposed for patients with permanent AF, RFC catheter ablation should be considered.

Procedure times for PVI using HIFU were long compared to RFC guided ablations. However, it has to be taken into consideration that HIFU BC is an evolving technique with room for further improvement. The new generation BC provides the possibility to advance a circular mapping catheter via a central lumen obviating the need for repeated deflation and inflation of the balloon in order to check for PVI. In fact, at our center 4 patients with paroxysmal AF (3 male, mean age 58 ± 14 years) were meanwhile treated with this new spiral mapping catheter (ProMap, ProRhythm, Inc.), which indeed allowed fast evaluation of ablation success. 15/16 PVs were isolated with the HIFU BC and procedure duration was markedly reduced to 127 ± 34 min. However, one patient experienced right sided PN palsy after sonication of the RSPV.

In the two published clinical trials 3 phrenic nerve injuries (PNI) were reported among 42 patients. All PNI occurred during sonication at the RSPV. At the RSPV the PN is in very close vicinity to the ostium, and if undersized balloons are used, the energy focus may include the PN [21]. Unfortunately, even after the introduction of CARTO based PN pace mapping one PN palsy occurred in the ProMap trial at our center. Thus, it seems to be  advisable to perform sonications at the RSPV (1) using oversized balloons and (2) after a  careful evaluation of the PN's anatomical course reassuring a distant course to the RSPV ostium.

A potential benefit of HIFU is the low thrombo-embolic potential. No such complications were observed in the clinical trials. However, the number of patients may be too low to asses the real clinical risk for thrombo-embolism using HIFU.

At present, we are aware of two complications involving the esophagus following HIFU treatment (one patient died due to an atrio-esophageal fistula, one patient recovered from esophageal laceration). In animal studies it was found that serial HIFU applications can cause esophageal lacerations if the balloon is positioned too close to the esophagus and if HIFU is delivered unabsorbed within the PV [22]. This usually occurs when the balloon is undersized and is positioned distally to the PV ostium. It is therefore advisable to use oversized balloons, sonicate in the LA (not inside the PV) and to maintain a balloon to esophagus distance of > 5mm. Monitoring the exact site of sonication and the location of the esophagus may best be possible using intravascular cardiac ultrasound (ICE) for visualization. The HIFU-device is presently being investigated in a randomized trial enrolling 240 patients in 20 Centers mostly in the USA. A similar protocol is evaluated by the ethical committee in 3 European Centers, which will also include the latest steerable HIFU catheter which has a small shaft of only 12 French.

## Conclusion

PVI using HIFU BC for patients with drug-refractory AF is feasible. Due to technical shortcomings the procedure times are still long and the patients with large PV ostia (>30mm) may not be ideal candidates for HIFU. Nonetheless, in clinical trials this investigational device has shown promising success rates similar to RFC catheter ablation. Therefore, it may be a valuable alternative to the conventional techniques in selected patients, but further clinical trials have to be initiated.

## Figures and Tables

**Figure 2:**
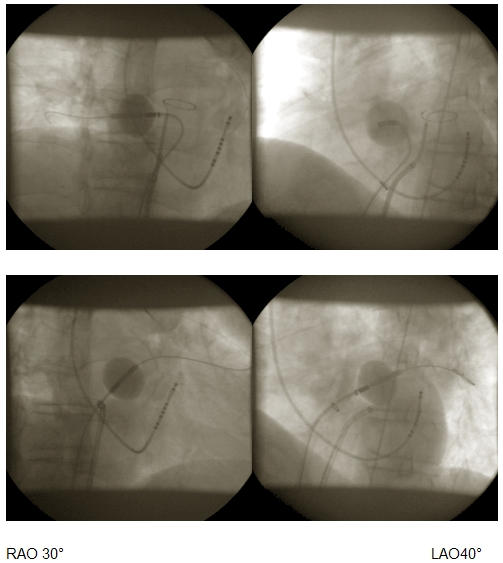
HIFU balloon catheter positioned at the right inferior PV (upper panel) and left superior PV (lower panel).
